# Surprising negative association between IgG1 allotype disparity and anti-adalimumab formation: a cohort study

**DOI:** 10.1186/ar3208

**Published:** 2010-12-27

**Authors:** Geertje M Bartelds, Els de Groot, Michael T Nurmohamed, Margreet HL Hart, Peter H van Eede, Carla A Wijbrandts, Jakob BA Crusius, Ben AC Dijkmans, Paul Peter Tak, Lucien Aarden, Gerrit J Wolbink

**Affiliations:** 1 Department of Rheumatology, Jan van Breemen Institute, Dr. Jan van Breemenstraat 2, 1056AB Amsterdam, The Netherlands; 2 Department of Immunopathology, Landsteiner Labaratory Sanquin Research, Plesmanlaan 125, 1066CX Amsterdam, The Netherlands; 3 Department of Rheumatology, VU University Medical Center, Postbus 7057, 1007MB Amsterdam, The Netherlands; 4 Department of Immunogenetics, Sanquin Diagnostic Services, Plesmanlaan 125, 1066CX Amsterdam, The Netherlands; 5 Department of Clinical Immunology and Rheumatology, Academic Medical Center/University of Amsterdam, Meibergdreef 9, 1105AZ Amsterdam, The Netherlands; 6 Department of Pathology Laboratory for Immunogenetics, VU University Medical Center, Postbus 7057, 1007MB Amsterdam, The Netherlands

## Abstract

**Introduction:**

The human monoclonal antibody adalimumab is known to induce an anti-globulin response in some adalimumab-treated patients. Antibodies against adalimumab (AAA) are associated with non-response to treatment. Immunoglobulins, such as adalimumab, carry allotypes which represent slight differences in the amino acid sequences of the constant chains of an IgG molecule. Immunoglobulins with particular IgG (Gm) allotypes are racially distributed and could be immunogenic for individuals who do not express these allotypes. Therefore, we investigated whether a mismatch in IgG allotypes between adalimumab and IgG in adalimumab-treated patients is associated with the development of AAA.

**Methods:**

This cohort study consisted of 250 adalimumab-treated rheumatoid arthritis (RA) patients. IgG allotypes were determined for adalimumab and for all patients. Anti-idiotype antibodies against adalimumab were measured with a regular radio immunoassay (RIA), and a newly developed bridging enzyme linked immunosorbent assay (ELISA) was used to measure anti-allotype antibodies against adalimumab. The association between AAA and the G1m3 and the G1m17 allotypes was determined. For differences between groups we used the independent or paired samples t-test, Mann-Whitney test or Chi square/Fisher's exact test as appropriate. To investigate the influence of confounders on the presence or absence of AAA a multiple logistic regression-analysis was used.

**Results:**

Adalimumab carries the G1m17 allotype. No anti-allotype antibodies against adalimumab were detected. Thirty-nine out of 249 patients had anti-idiotype antibodies against adalimumab (16%). IgG allotypes of RA patients were associated with the frequency of AAA: patients homozygous for G1m17 had the highest frequency of AAA (41%), patients homozygous for G1m3 the lowest frequency (10%), and heterozygous patients' AAA frequency was 14% (*P *= 0.0001).

**Conclusions:**

An allotype mismatch between adalimumab and IgG in adalimumab-treated patients did not lead to a higher frequency of AAA. On the contrary, patients who carried the same IgG allotype as present on the adalimumab IgG molecule, had the highest frequency of anti-adalimumab antibodies compared to patients whose IgG allotype differed from adalimumab. This suggests that the allotype of adalimumab may not be highly immunogenic. Furthermore, patients carrying the G1m17-allotype might be more prone to antibody responses.

## Introduction

**Figure 1:**
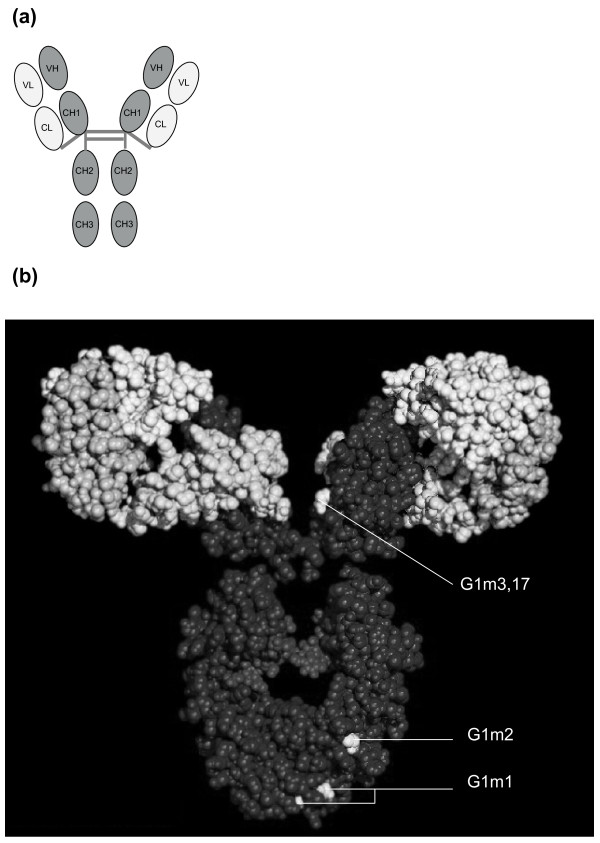
**Basic immunoglobulin structure and IgG1 allotypes**. **(a)**. Basic immunoglobulin structure. CH1, 2 and 3 are the constant heavy chains. CL is the constant light chain. VH is the variable heavy chain and VL the variable light chain which together form the variable domain of the immunoglobulin, a specific antigen binding site, also referred to as the idiotype. **(b)**. IgG1 allotypes [7]. The white residues in the constant parts are those residues which differ by allotype in human IgG1. There is a Lys (G1m17) for Arg (G1m3) change at codon 214 in the CH1 domain, an Asp 356 Leu 358 (G1m1) for Glu 356 Met 358 (nG1m1) in the CH3 domain and a Gly 431 (G1m2) for Ala (nG1m2) also in the CH3 domain. The nG1m1 and nG1m2 are not "true" allotypes because these amino acid residues are present in other IgG subclasses and are not expected to be immunogenic in the individual.

Treatment with monoclonal antibodies (mAbs) is known to induce anti-mAb antibodies, leading to a diminished treatment response [1-5]. The general structure of all antibodies is very similar; it consists of a constant and a variable region, the variable region determines the idiotype. The anti-adalimumab antibodies (AAA) measured in previous studies are anti-idiotype antibodies, directed against the idiotype of adalimumab [1,6]. The constant region is almost identical in all antibodies of the same isotype, but differs in antibodies of different isotypes (for example, IgA, IgM, IgG, IgE, IgD). However, within an immunoglobulin of a certain isotype, allotypes represent slight differences in the amino acid sequences of the constant heavy or light chains of different individuals (Figure 1) [7]. There are different allotypes for IgG1, IgG2 and IgG3 and no allotypes have been found for IgG4. Allotypes are inherited in a codominant Mendelian way, in fixed combinations called haplotypes. Allotypes expressed on the constant region of IgG heavy chain are designated as Gm (Genetic markers) together with the subclass. Allotypes of heavy γ1 chains are defined as G1m allotypes, allotypes of heavy γ2 chains as G2m allotypes, and of heavy γ3 chains as G3m allotypes. The Gm system is unique in its ability to characterize human populations by specific sets of haplotypes. Specific Gm haplotypes are found in African, Caucasian and Mongoloid populations. In a Caucasian population the G1m1,17 (or G1m(a,z)) allotype is much less frequent (0.15 to 0.35) than G1m3 (or G1m(f)) (0.65 to 0.85) [8]. Therefore, serologically defined allotypes differ widely within and between population groups [9].

Allotypic markers can therefore differ between individuals and immunoglobulins with certain allotypes can be immunogenic when injected into individuals whose immunoglobulins lack the allotype. Treatment with monoclonal antibodies with a certain allotype can lead to the formation of anti-allotype antibodies. The allotypes of a panel of licensed mAbs was determined and adalimumab expresses the G1m1,17-allotype [9]. The risk to provoke antibody responses as a result of allo-immunization has been described in a review [9]. MAb treatment of patients may lead to both allo-immunization and/or xeno-immunization that result in antisera that may recognize isotypic, allotypic and idiotypic epitopes.

The association between anti-infliximab antibodies and immunoglobulin allotypes was recently investigated [10]. Infliximab expresses the G1m1,17-allotype, the hypothesis of this study was that patients without the G1m1,17-allotype were more likely to develop anti-infliximab antibodies. However, no association was found between the patients' allotypes and the presence or concentration of anti-infliximab antibodies. The authors pose the question whether this would also be the case for humanized or fully human antibodies, because in chimeric antibodies the murine variable domain could dominate the antibody response. This might not be the case for humanized or human monoclonal antibodies.

In this study we investigated whether an IgG allotype mismatch between adalimumab- and adalimumab-treated patients is associated with a higher frequency of AAA.

## Materials and methods

### Patients

All 250 consecutive unrelated RA patients were included in a prospective observational cohort at the outpatient clinics of the Departments of Rheumatology of the Jan van Breemen Institute and the Academic Medical Center in Amsterdam. All patients fulfilled the American College of Rheumatology 1987 revised criteria for RA [11], and had active disease, indicated by a disease activity score in 28 joints (DAS28) of ≥3.2 despite earlier treatment with two disease modifying anti-rheumatic drugs (DMARDs) including methotrexate (MTX) at a dosage of 25 mg weekly or at the maximal tolerable dosage, according to the Dutch consensus statement on the initiation and continuation of TNF blocking therapy in RA [12]. Patients were treated with either adalimumab and concomitant DMARD therapy, or adalimumab monotherapy. All patients used adalimumab 40 mg subcutaneously every other week. In patients with an inadequate response as judged by the treating rheumatologist, the dosing frequency of adalimumab could be increased to 40 mg per week. The study was approved by the Medical Ethics Committee of the Slotervaart Hospital, BovenIJ Hospital, the Jan van Breemen Institute, and the Academic Medical Center/University of Amsterdam. All patients gave written informed consent.

### Clinical response to adalimumab

Disease activity was assessed at baseline and after 28 weeks of therapy using the DAS28 score. Clinical response was assessed by the decrease in DAS28 score (ΔDAS28) and the European League Against Rheumatism (EULAR) response criteria [13].

### The measurement of human IgG1 allotypes

The most prevalent allotypes of the G1m system were measured: G1m1, G1m3 and G1m17.

Immunoglobulin allotypes were determined by an enzyme-linked immunosorbent assay (ELISA) using specific antibodies against Gm markers. All incubations were at room temperature. Plates were coated for two hours with 0.5 μg/ml of a mouse monoclonal antibody to human IgG1 (MH161-1, Sanquin, Amsterdam, The Netherlands). After washing, the plates were incubated for one hour with serum of interest, and diluted 1:1000 in PTG buffer (PBS, 0.2% gelatine, 0.02% Tween). Afterward washing plates were incubated for one hour with allotype-specific biotinylated monoclonal antibodies. For that purpose we used anti-G1m3 (%A1), anti-G1m17 (5F10) and anti-G1m1 (MG102-A2 at 10 ng/ml (Sanquin). After washing, the plates were incubated with polymerTsed streptavidin-horseradish peroxidase (poly-HRP). Non-bound streptavidin-poly-HRP was removed by washing and the amount of bound streptavidin was measured by incubating the plates with tetramethylbenzidine (TMB), the substrate for HRP. The reaction was stopped with H_2_SO_4_. Absorption at 450 nm was determined in a microtiter plate reader. The results of the unknown sera were compared with the sera with known allotypes.

### Measurement of antibodies against adalimumab

Serum samples were collected at baseline and just prior to an injection with adalimumab after 4, 16 and 28 weeks. The presence of AAA was determined at all time points between baseline and 28 weeks. AAA were detected with a radio immunoassay (RIA). One micro litre of serum diluted in PBS/0.3% bovine serum albumin (BSA) (PA buffer) was incubated over night with 1 mg Sepharose-immobilized protein A (GE Healthcare, Chalfont, St. Giles, UK) in a final volume of 800 μl. Subsequently the samples were washed with PBS 0.005% Tween and specific ADA binding was detected by o/n incubation with 20.000 dpm (approximately 1 ng) 1,25I labeled F(ab)2 adalimumab diluted in Freeze buffer (Sanquin). The unbound label was removed by washing, and protein A bound radioactivity was measured. When binding was higher than 25% of the input, sera were further titrated. Antibody levels were compared to a standard serum containing anti-drug antibody levels and expressed in arbitrary units (AU). One AU corresponds to approximately 12 ng. The mean cut-off value was set at 12 AU/ml which was derived from 100 healthy donors. Assay specificity was demonstrated by the absence of AAA in 25 sera containing high-titres anti-infliximab antibodies from patients not treated with adalimumab. In the assays we did not find cross reactivity. Recently, patient sera were tested in a bioassay, which confirmed the specificity and validity of the RIA [14]. Patients were defined as positive for anti-adalimumab antibodies if titres were above 12 AU/ml on at least one occasion, in combination with serum adalimumab levels below 5.0 mg/L. All baseline samples before the start of treatment were negative.

### Measurement of anti-allotype antibodies against adalimumab/infliximab

We searched for anti-allotype antibodies using a two-sided assay/bridging ELISA. Sera were tested for their capacity to make a bridge between coated adalimumab and biotinylated adalimumab. To that end plates were coated with 0.5 μg/ml adalimumab in phosphate buffered saline. After washing, the plates were incubated with patient sera. After washing, the plates were incubated with biotinylated adalimumab at 5 ng/ml. After washing, the plates were incubated with streptavidin-poly-HRP and developed as described above. All sera that were positive in the RIA were also positive in this bridging ELISA. Adalimumab and infliximab have the same allotype (G1m1,17). We reasoned that if sera were positive due to anti-allotype antibodies, these sera should also be positive if adalimumab was replaced by infliximab. Therefore, sera were also tested for their capacity to bridge infliximab with adalimumab and adalimumab with infliximab. As controls we used rabbit anti-adalimumab-idiotype, rabbit anti-infliximab-idiotype and a monoclonal antibody to human IgG (MH16-1).

### Statistical analysis

For statistical analysis SPSS version 16.0 (SPSS Inc., Chicago, Illinois, USA) was used. We chose to analyze the association among AAA and the G1m3 and the G1m17 allotypes at codon 214 (Figure 1), since these allotypes correspond with a single amino acid change and haplotype construction is not required. For differences between groups we used the independent or paired samples t-test, Mann-Whitney test or Chi square/Fisher's exact test as appropriate. To investigate the influence of confounders on the presence or absence of AAA, a multiple logistic regression-analysis was used. Variables considered potential confounders were chosen from all available baseline variables and were determined for every analysis specifically, based on differences between groups included in the analysis. Variables were included in the regression model as confounders if the beta changed 10% or more after inclusion of the variable in the model. The threshold for significance was set at *P *< 0.05. To analyze clinical response we used the last observation carried forward for patients who stopped treatment due to non-response or adverse events, and for patients who had received increased adalimumab dosing frequency.

## Results

### Patient characteristics

Patient characteristics are shown in Table 1. Of the 250 patients enrolled in the study, six (2%) discontinued adalimumab treatment after four weeks of therapy, and 16 (6%) stopped treatment after Week 16. Ten patients (4%) stopped due to treatment failure, nine (4%) because of adverse events and three (1%) were lost to follow-up. Twenty-one patients (8%) had an increased dosing frequency before 28 weeks to 40 mg adalimumab per week; in these patients the last DAS28 before dose increase was carried forward to 28 weeks.

**Table 1:** Demographic and clinical characteristics at baseline

	Total population
	*n *= 250
Demographics	
Age, years	52 ± 13
Female, no. (%)	197 (79)

DMARD therapy	
Prior DMARDs (no.)	3.4 ± 1.6
Methotrexate use, no. (%)	199 (80)
Methotrexate dose (mg/wk)	23 (15 to 25)
Prednisone use, no. (%)	82 (33%)
Prednisone dose (mg/day)	7.5 (5 to 10)

Disease status	
Disease duration (years)	8 (4 to 17)
Rheumatoid factor positive, no. (%)	179 (72)
Erosive disease, no. (%)	194 (78)
Erythrocyte sedimentation rate (mm/h)	30 ± 23
C-reactive protein (mg/dl)	11 (5 to 24)
DAS28	5.2 ± 1.2

Mean values ± SD, median and interquartile range, or percentages are shown.

DAS28, Disease Activity Score in 28 joints; DMARD, diseases modifying anti rheumatic drug.

### Clinical response

The mean DAS28 after 28 weeks of adalimumab therapy decreased from 5.2 ± 1.2 at baseline to 3.7 ± 1.5 (*P *= 0.0001). There were 63 (25%) non-responders, 105 (42%) moderate responders, and 82 (33%) good responders according to the EULAR response criteria.

### Association between allotypes and anti-adalimumab antibodies

Thirty-nine out of 249 patients had antibodies against adalimumab (16%); in one patient AAA could not be determined. Patients without AAA had a significantly greater DAS28 improvement than patients with AAA (ΔDAS28 = 1.7 versus ΔDAS28 = 0.5, *P *= 0.0001).

Adalimumab and infliximab have the same allotype G1m1,17 [9,10]. Nevertheless we observed that all sera positive in the assay for antibodies to adalimumab (hence the adalimumab-adalimumab combination) were negative in the assay for anti-infliximab (the infliximab-infliximab combination) as well as in the assay where adalimumab was combined with infliximab. The anti-IgG was strongly positive in all three assays. Our conclusion is that these patients do not make anti-allotype antibodies and that all AAA's are due to anti-idiotypic antibodies.

There was a significant association between the G1m3 and G1m17 allotypes and antibodies against adalimumab (Table 2). After adjustment for MTX dose in logistic regression the carriage of more G1m17 alleles was significantly associated with a higher frequency of antibodies against adalimumab (*P *= 0.0001; OR = 2.639; 95% CI = 1.608 to 4.332). Baseline characteristics for the three groups with G1m3 and G1m17 allotypes are shown in Table 3.

**Table 2:** Association between G1m3 and G1m17 allotypes and antibodies against adalimumab

G1m phenotype	AAA -	AAA +
3,3	108 (90%)	12 (10%)
3,17	83 (86%)	14 (14%)
17,17	19 (59%)	13 (41%)

AAA -, anti-adalimumab antibodies negative; AAA +, anti-adalimumab antibodies positive.

Patient numbers and corresponding percentages are shown. *P *= 0.0001 for the whole table.

**Table 3:** Demographic and clinical characteristics at baseline

	G1m phenotype		
	3,3	3,17	17,17
	*n *= 120	*n *= 98	*n *= 32
Demographics			
Age, years	53 ± 14	53 ± 12*	48 ± 12*
Female, no. (%)	95 (79)	74 (76)	28 (88)
			
DMARD therapy			
Prior DMARDs (no.)	3.5 ± 1.6	3.4 ± 1.7	3.3 ± 1.6
Methotrexate use, no. (%)	95 (79)	81 (83)	23 (72)
Methotrexate dose (mg/wk)	21 (15 to 25)	25 (15 to 25)	25 (17.5 to 25)
Prednisone use, no. (%)	39 (33)	31 (32)	12 (38)
Prednisone dose (mg/day)	7.5 (5 to 10)	5 (5 to 7.5)	10 (5 to 10)

Disease status			
Disease duration (years)	10 (4 to 17)	11 (3 to 16)	7 (3 to 17)
Rheumatoid factor positive, no. (%)	80 (67)*	70 (71)	29 (91)*
Erosive disease, no. (%)	90 (75)	76 (78)	28 (88)
Erythrocyte sedimentation rate (mm/h)	29 ± 23	31 ± 25	28 ± 17
C-reactive protein (mg/dl)	11 (4 to 24)	10 (6 to 23)	14 (6 to 31)
DAS28	5.1 ± 1.2	5.3 ± 1.2	5.2 ± 1.0

DAS28, Disease Activity Score in 28 joints; DMARD, diseases modifying anti rheumatic drug.

*There was a significant difference between patients with the 3,17-allotype and the 17,17-allotype for age (*P *= 0.020), and between both homozygous groups 3,3 and 17,17 for rheumatoid factor positivity, no. (*P *= 0.007).

## Discussion

Our hypothesis was that a mismatch between the allotype of adalimumab, G1m1,17, and the allotypes of the IgG of adalimumab treated RA patients would be associated with a higher frequency of anti-adalimumab antibodies. This was not the case. The first explanation for this lack of association could be that neither of the assays we used was able to detect anti-allotype antibodies. Our RIA for the detection of AAA is designed to detect anti-idiotype antibodies. In this assay a solution containing pepsine treated polyclonal IgG Freeze buffer is added, as a result anti-allotype antibodies are not detected. However, without Freeze buffer anti-allotype antibodies also were not detected. No anti-allotype antibodies were detected with the bridging ELISA. It might be possible that the bridging ELISA was not able to detect anti-allotype antibodies, due to low titers, epitope masking or steric hindrance. Another explanation could be that anti-allotype antibodies are not developed or that the quantity of the anti-allotype antibody response is not large enough to be detected. The allotypes of adalimumab may not be highly immunogenic, and could be only a minor antigen compared to the idiotype of adalimumab. Patients who were homozygous for G1m3, for whom the allotype of adalimumab theoretically would be immunogenic, had a clinical response that did not differ from patients who carried the G1m17-allotype after adjustment for having anti-idiotype antibodies against adalimumab (data not shown). This suggests that if anti-allotype antibodies had developed, their clinical relevance would be nil.

Our hypothesis could not be confirmed, but the results showed an unexpected association between allotypes and AAA: RA patients whose IgG1 was homozygous for the same allotype as adalimumab, the G1m17-allotype, had the highest frequency of AAA compared to G1m3 homozygotes or heterozygotes. This suggests that the frequency of AAA has no relation with the possible immunogenicity of the allotype of adalimumab, but is more likely explained by patient-related genetic factors. A selective force behind the distribution and inheritance of allotypes may have been the association between immunoglobulin allotypes and the specific antibody responses to pathogens, resulting in differential immunity to infectious diseases [15]. For numerous infectious diseases an association has been found between immunoglobulin allotypes and (the level of) antibody response [15]. There are several studies in which the G1m1,17-allotype or a haplotype containing this allotype was associated with a stronger immune response compared to individuals with the G1m3-allotype. For example, systemic sclerosis patients homozygous for the G1m3 allele were 60% less likely to be seropositive for IgG antibodies against cytomegalovirus than patients homozygous for the G1m17 allele or the heterozygotes [16]. Hepatitis C virus (HCV) infected patients with the Gm1,17 5,13 phenotype within an African American population had two-fold higher median antibody titres against E1 and E2 envelope glycoproteins, HCV epitopes than those who lacked this phenotype [17]. In a study on the association of allotypes with antibodies against MUC1, a tumor-associated antigen, gastric cancer patients with the phenotype Gm3 23 5, 13 had lower anti-MUC1-IgG levels compared to patients without this phenotype [18].

Patients with the G1m3 phenotype not only had AAA significantly less often, but were also less often positive for rheumatoid factor (Table 3). This also contributes to the hypothesis that allotypes are associated with specific antibody responses. Individuals with a G1m1,17-allotype might be more prone to antibody responses than individuals with the G1m3-allotype. No conclusive data are available on how allotypes could influence immune response, albeit several possibilities are mentioned [15]. The locus or loci responsible for the association with immune response may not be the Gm system itself but may reflect linkage disequilibrium with other polymorphisms of the constant region genes or with specific variable region genes. There is evidence for a genetic predisposition to the formation of antibodies. Previously, we showed that interleukin-10 (IL10) polymorphisms were associated with anti-adalimumab antibody formation in RA [19]. However, we did not find an association between IL10 polymorphisms and IgG allotypes (data not shown).

## Conclusions

To our knowledge this is the first study examining the association between G1m allotypes and immunogenicity against adalimumab. Our findings suggest that the allotype is not a dominant antigen of adalimumab. Albeit we have to take into account that we did not find anti-allotype antibodies. Interestingly, our data show that anti-adalimumab antibody formation occurred more often in RA patients with the G1m17-allotype than in RA patients without this allotype, which indicates a role for genetic factors. Patients carrying this allotype might be more prone to antibody responses. However, these results should be replicated in larger study populations with a representative variation in allotypes in order to draw firm conclusions.

## Abbreviations

AAA: anti-adalimumab antibodies; AU: arbitrary units; BSA: bovine serum albumin; DAS28: disease activity score in 28 joints; DMARDs: disease modifying anti-rheumatic drugs; ELISA: enzyme linked immunosorbent assay; EULAR: European League Against Rheumatism; Gm: Genetic marker; HCV: hepatitis C virus; HRP: horseradish peroxidase; IL: interleukin; mAbs: monoclonal antibodies; MTX: methotrexate; RA: rheumatoid arthritis; RIA: radio immunoassay; TMB: tetramethylbenzidine; TNF: tumour necrosis factor.

## Competing interests

BD and PT are members of the advisory board of Abbott, and BD, PT and MN have received honoraria for lectures. PT has served as a consultant to Abbott, Amgen, Centocor, Schering-Plough, UCB, and Wyeth. BD received research grants from Schering-Plough, Wyeth and Abbott. The other authors declare that they have no competing interests.

## Authors' contributions

GW had full access to all the data in the study and had final responsibility for the decision to submit for publication. GB, EG, MN, MH, BC, LA and GW participated in the study design. GB, MN, CW, PT and GW were involved in the acquisition of the data. GB took part in the data analysis and EG and MH in carrying out the immunoassays. GB, LA and GW participated in the interpretation of the data. All authors participated in the preparation of the manuscript and saw and approved the final version.
